# Occurrence of High-Risk Clonal Lineages ST58, ST69, ST224, and ST410 among Extended-Spectrum β-Lactamase-Producing *Escherichia coli* Isolated from Healthy Free-Range Chickens (*Gallus gallus domesticus*) in a Rural Region in Tunisia

**DOI:** 10.3390/genes14040875

**Published:** 2023-04-06

**Authors:** Saloua Benlabidi, Anis Raddaoui, Sana Lengliz, Sarah Cheriet, Paul Hynds, Wafa Achour, Taoufik Ghrairi, Mohamed Salah Abbassi

**Affiliations:** 1 Institute of Veterinary Research of Tunisia, University of Tunis El Manar, Tunis 1006, Tunisia; saloua.benlabidi@gmail.com (S.B.); lengliz_sana@yahoo.fr (S.L.); sarahcheriet5@gmail.com (S.C.); 2 Laboratory of Neurophysiology Cellular Physiopathology and Biomolecule Valorisation LR18ES03, Faculty of Sciences of Tunis, University Tunis El Manar, Tunis 1068, Tunisia; taoufik.ghrairi@fst.utm.tn; 3 Laboratory Ward, National Bone Marrow Transplant Center, Tunis 1006, Tunisia; anis.raddaoui@yahoo.fr (A.R.); wafaachour@gmail.com (W.A.); 4 Laboratory of Materials, Molecules and Application LR11ES22, Preparatory Institute for Scientific and Technical Studies, University of Carthage, Tunis 1054, Tunisia; 5 Environmental Sustainability and Health Institute (ESHI), Technological University Dublin, D07 H6K8 Dublin, Ireland; hyndsp@tcd.ie; 6 Research Laboratory ‘Antimicrobial Resistance’ LR18ES39, Faculty of Medicine of Tunis, University of Tunis El Manar, Tunis 1006, Tunisia

**Keywords:** free-range chickens, ESBL, *Escherichia coli*, CTX-M-1, *mcr*-2, high-risk clone

## Abstract

Antimicrobial-resistant *Escherichia coli* isolates have emerged in various ecologic compartments and evolved to spread globally. We sought to (1.) investigate the occurrence of ESBL-producing *E. coli* (ESBL-Ec) in feces from free-range chickens in a rural region and (2.) characterize the genetic background of antimicrobial resistance and the genetic relatedness of collected isolates. Ninety-five feces swabs from free-range chickens associated with two households (House 1/House 2) in a rural region in northern Tunisia were collected. Samples were screened to recover ESBL-Ec, and collected isolates were characterized for phenotype/genotype of antimicrobial resistance, integrons, and molecular typing (pulsed-field gel electrophoresis (PFGE) and multilocus sequence typing (MLST)). Overall, 47 ESBL-Ec were identified, with the following genes detected: 35 *bla*_CTX-M-1,_ 5 *bla*_CTX-M-55_, 5 *bla*_CTX-M-15_, 1 *bla*_SHV-2,_ and 1 *bla*_SHV-12_. Resistance to fluoroquinolones, tetracycline, sulfonamides, and colistin was encoded by *aac(6′)*-Ib-*cr* (*n* = 21), *qnr*B (*n* = 1), and *qnr*S (*n* = 2); *tet*A (*n* = 17)/*tet*B (*n* = 26); *sul*1 (*n* = 29)/*sul*2 (*n* = 18); and *mcr*-2 (*n* = 2) genes, respectively. PFGE and MLST identified genetic homogeneity of isolates in House 1; however, isolates from House 2 were heterogeneous. Notably, among nine identified sequence types, ST58, ST69, ST224, and ST410 belong to pandemic high-risk clonal lineages associated with extrapathogenic *E. coli*. Minor clones belonging to ST410 and ST471 were shared by chickens from both households. The virulence genes *fyuA, fimH, papGIII,* and *iutA* were detected in 35, 47, 17, and 23 isolates, respectively. Findings indicate a high occurrence of ESBL-Ec in free-range chickens and highlight the occurrence of pandemic zoonotic clones.

## 1. Introduction

Antimicrobial resistance has emerged as a critical public health challenge globally. It has been associated with hospital (nosocomial) environments; however, antimicrobial-resistant bacteria (ARB) are increasingly reported from livestock, animal-derived food products, companion animals, wild animals, aquatic environments, and agricultural and nonagricultural soils [1,2,3] Many opportunistic and pathogenic Enterobacterales species, including *Escherichia coli*, have been reported as important reservoirs for multiple genes encoding resistance to clinically relevant antibiotics, such as β-lactams, fluoroquinolones, and polymixins (colistin) [4,5]. The major mechanism for β-lactam resistance is the production of β-lactamase enzymes, particularly extended-spectrum β-lactamases (ESBLs), AmpC β-lactamases (AmpC), and carbapenemases (CAP) [5,6]. These enzymes are typically plasmid-borne, thus enabling rapid spread within strains belonging to the same or different Enterobacterales genera [7,8,9]. ESBL-producing Enterobacteriales, including *E. coli*, are currently listed among the top twelve critical drug-resistant threats by the Centers for Disease Control and Prevention (CDC) [10]. Additionally, they are included in the World Health Organization (WHO) priority list of antibiotic-resistant bacteria to guide research, discovery, and development of new antibiotics [11].

Historically, ESBLs were primarily derived from genes for the narrow-spectrum β-lactamases (TEM-1, TEM-2, or SHV-1); however, since the early 2000s, the new CTX-M type has emerged, dramatically changing the epidemiology of ESBL enzymes, with CTX-M now the dominant enzymes worldwide (Bush and Bradford 2020). It is worth noting that, currently, approximately 243 TEM variants have been described, although not all of them are ESBLs (https://www.ncbi.nlm.nih.gov/pathogens/refgene/#TEM, accessed on 25 January 2022). The same is true for SHV enzymes; indeed, to date, 228 SHV sequence variants have been detected, although not all have been functionally characterized to determine whether they possess the ESBL phenotype (https://www.ncbi.nlm.nih.gov/pathogens/ isolates#/refgene/SHV, accessed on 25 January 2022). On the other hand, to date, at least 263 CTX-M variants, which are all ESBLs, have been identified (https://www.ncbi.nlm.nih.gov/pathogens/refgene/#CTX, accessed on 25 January 2022). The reported genetic linkage between genes encoding these ESBL enzymes with genes encoding resistance to other clinically relevant antibiotics, including aminoglycosides, tetracyclines, sulfonamides, trimethoprim, phenicols, and colistin, represents a very significant concern [12,13]. Traditionally, it was believed that excessive use of antibiotics in healthcare settings and livestock represented the major drivers for selection and transmission of these strains. However, several recent studies have documented their occurrence in wild animals, animals not having undergone antimicrobial therapies, and environments characterized by limited anthropogenic impacts or inputs [1,3,8,14]. Several hypotheses have been proposed to explain these occurrences, with perhaps the most likely being environmental spread of ARB and antimicrobial resistance genes (ARGs) via hospital effluents, municipal wastewater treatment plants (WWTPs), and the use of animal manures as agricultural fertilizers [14]. Additionally, wild animals residing in the vicinity of human conurbations and livestock-based farms have been shown to represent major vectors of ARB and ARGs, contributing to their spread across large geographic area, particularly by wild birds [1,8,15]. 

*E. coli* is a commensal bacterium of the lower intestine of warm-blooded animals and is capable of causing several human infections, including acute gastrointestinal illness (AGI), urinary tract infections, and septicemia [16]. Likewise, *E. coli* may cause several acute infections in agricultural animals, including colibacillosis in poultry and mastitis in cattle [16,17,18]. Since *E. coli* is considered a key contributor in the environmental spread of antibiotic resistance [19], it has been widely employed as an indicator for monitoring antibiotic resistance in food products and water [20,21]. To date, a paucity of scientific literature exists regarding the presence of ESBL-producing *E. coli* in “backyard”/free-range poultry or swine [22,23,24]. However, studies have shown that the close proximity between owners and their livestock may potentially increase transmission of ARB or ARGs from humans to animals and vice versa. In Tunisia, while previous studies have reported a high prevalence of ESBL-producing *E. coli* from industrial avian farms [12,25,26], to the best of the authors’ knowledge, no study has investigated the occurrence of ESBL-producing *E. coli* isolates in free-range chickens in rural ecosystems. 

Over the past decade, many organizations and governments have adopted the “One Health” approach to assist in combating the emergence and spread of antimicrobial resistance, recommending continuous and widespread monitoring of antimicrobial resistance across a diverse range of ecosystems. Accordingly, the current study aimed to investigate the occurrence of ESBL-producing *E. coli* in feces from free-range chickens (poultry that are raised by free-range farming) in a rural region in northern Tunisia. Collected isolates were characterized via identification of genes encoding antibiotic resistance and virulence factors, integrons, and their phylogenetic grouping, in addition to genetic relatedness via pulsed-field gel electrophoresis (PFGE) and multilocus sequence typing (MLST).

## 2. Materials and Methods

### 2.1. Sampling Sites

The presence of ESBL-producing *Enterobacteriaceae* was investigated using a sample of 95 feces swabs from free-range chickens (*Gallus gallus domesticus*) owned by two families (House 1: *n* = 40; House 2: *n* = 55) in a rural region of northern Tunisia, collected during the period of February–March 2019. The two houses were randomly selected in one of the principal Tunisian regions of wheat crops. The region is under limited anthropogenic impacts or inputs and there were no rivers or riverines (which might contain and bring resistant bacteria or genes encoding antimicrobial resistance). Chickens were raised for supplementary income and/or household consumption, with the households located approximately 300 m apart in an agricultural area. Both subsamples were raised as free-roaming birds and fed locally available grain (wheat and barley) and kitchen scraps. Both families also reared cattle (1–3 per family), ewes (7–15 per family), domestic pets (dogs and cats), and donkeys, with chickens reared in close proximity to these animals. According to both owners, chickens did not previously receive antimicrobial agents for any purpose (therapy or growth promotion). 

### 2.2. Feces Swab Samples and Bacterial Identification 

The feces swabs were collected from each bird, stored at 4 °C, and transferred to the laboratory within 24 h, with cold chain constantly maintained. Approximately 5 mL of Brain Heart Infusion (BHI) (Oxoid Ltd., Basingstoke, UK) was added to each sample, followed by incubation at 37 °C for 2 h. Subsequently, 1 mL of enriched suspension was streaked onto tryptone bile X-glucuronide agar (TBX agar; Oxoid Ltd., Basingstoke, UK) plates supplemented with 2 mg/L of cefotaxime (Sigma-Aldrich, Munich, Germany) and incubated overnight at 37 °C [26,27] for recovery of cefotaxime-resistant *Enterobacteriaceae* (potential producers of ESBL and acquired pAmpC). One colony per sample (bird) was randomly selected, identified using API 20E (Bio-Mérieux. La Balme, les Grottes, France), and confirmed as *E. coli* via species-specific PCR amplification of the *uid* gene, encoding for β-glucuronidase (primers used: *uid*-F: 5′ ATCACCGTGGTGACGCATGTCGC 3′; *uid*-R: 50 CACCACGATGCCATGTTCATCTGC 3′) [28].

### 2.3. Antimicrobial Susceptibility Testing

Antimicrobial susceptibility testing was undertaken using the disk diffusion method on Mueller–Hinton agar (Oxoid Ltd.) plates, according to Clinical Laboratory Standard Institute guidelines [29]. The Minimal Inhibitory Concentration (MIC) for colistin was determined by the broth microdilution method [29]; isolates exhibiting an MIC ≥ 4 µg/mL were considered colistin-resistant. The double-disk synergy test (DDST) between amoxicillin/clavulanic acid (AMC, 20/10 µg) and ceftazidime (CAZ, 30 µg), aztreonam (ATM, 30 µg), and cefotaxime (CTX, 30 µg) was used to detect ESBL production (CLSI 2017). *E. coli* ATCC 25922 and *Klebsiella pneumoniae* ATCC 700603 were used as ESBL-negative and positive reference strains, respectively, with isolates considered multidrug-resistant (MDR) when found to be resistant to three or more antibiotics from different families [30]. 

### 2.4. Resistance Genotypes and Occurrence of Integrons

Genomic DNA was extracted from each isolate using the boiling method [25] and used as the DNA template for all PCR reactions. The presence of ESBL genes *bla*_TEM_, *bla*_SHV,_ and *bla*_CTX-M_ groups (CTX-M-1, CTX-M-2, CTX-M-8, and CTX-M-9) and OXA-1 and OXA-10 were investigated by PCR reactions [28,31,32]. *bla*_CTX-M_ and *bla*_SHV_ amplicons were sequenced and analyzed using BLAST software version BLAST+(2.11.0) (http://blast.ncbi.nlm.nih.gov/Blast.cgi, accessed on 25 January 2022) to determine the ESBL variant. 

The following genes encoding resistance to non-β-lactams were investigated by PCR: tetracycline (*tet*A, *tet*B and *tet*C) [33,34], trimethoprim-sulfamethoxazole (*sul*1, *sul*2, and *sul*3) [35,36,37], fluoroquinolones (plasmid-mediated quinolone resistance, PMQR) (*qnrA*, *qnrB*, *qnrD*, *qnrS*, *aac(6′)-*Ib, and *qepA*) [38,39,40,41,42], and colistin (*mcr*-1 to *mcr*-5) [43,44]. Amplicons corresponding to *mcr* genes were sequenced, and sequences were compared with those included in the GenBank database. The presence of class 1 and 2 integrons and the 3′- conserved region (*qac*∆E-*sul*1) of class 1 integrons was examined by PCR [34]. Positive controls strains from our collection were included in all PCR reactions [12,26,27].

### 2.5. Detection of Genes Encoding Virulence Factors

The detection of virulence factors commonly found in pathogenic *E. coli* (*fim*H, *iut*A, *fyu*A, *pap*G allele III, *hly*A, *cnf1*) were identified via PCR using previously published methods [45,46].

### 2.6. Phylogrouping of E. coli isolates Genetic Relatedness by Pulsed-Field Gel Electrophoresis (PFGE) and Multilocus Sequence Typing (MLST)

Phylogenetic groups (A, B1, B2, C, D, E, F, and *Escherichia* cryptic clade I) were identified using the phylogroup assignment method of Clermont et al [47]. To assess the clonality of collected isolates, genomic DNA was prepared, digested by *Xba*I enzyme and analyzed by PFGE, as reported previously [34]. DNA macrorestriction patterns (pulsotypes) were visually analyzed and interpreted according to Tenover et al [48]. Dendrogram for the main phylotypes was performed using GelJ software (version 1.0) [49], based on the Dice similarity coefficient, and clustering by the unweighted pair group method with arithmetic means (UPGMA method) [50]. Bands were selected by the software and then manually annotated. In addition, representative *E. coli* isolates belonging to relevant PFGE profiles and harboring specific *bla* and *mcr* genes or untypeable by PFGE were studied by MLST using PCR amplification and sequencing of seven conserved housekeeping genes (*adk*, *fumC*, *gyrB*, *icd*, *mdh*, *purA*, and *recA*) [51]. To determine the specific allele combination and sequence type (ST), all amplicons sequences were compared with MLST databases (http://enterobase.warwick.ac.uk/species/ ecoli/allele_st_search, accessed on 25 January 2022).

## 3. Results

### 3.1. Occurrence of ESBL-Producing E. coli and Antibiotic Susceptibilities of Isolates

Overall, based on one presumptive *E. coli* isolate *per* bird, which was randomly selected and confirmed to the species level by PCR, 47 (49.4%) sampled chickens were colonized by ESBL-producing *E. coli* isolates. More specifically, 29 (29/40; 72.5%) and 18 (18/55; 32.7%) ESBL-producing isolates were collected from House 1 and House 2, respectively. All isolates were resistant to at least one non-β-lactam antibiotic, with the majority being characterized as MDR, with isolates primarily resistant to nalidixic acid, tetracycline, and trimethoprim–sulfamethoxazole (Table 1). Nalidixic acid resistance was detected in 25 and 13 isolates from House 1 and 2, respectively, with ciprofloxacin resistance concurrently identified in 17 of the 38 nalidixic-acid-resistant isolates. Colistin resistance was observed in three and ten isolates from chickens from House 1 and 2, respectively. Colistin MICs varied from 4 µg/mL to 16 µg/mL. Resistance to carbapenems (imipenem, meropenem, and ertapenem) was not observed.

### 3.2. Genes Encoding ESBL Enzymes and Non-β-Lactam Antibiotics

PCR analyses showed a predominance of *bla*_CTX_-type genes; *bla*_CTX-M-1_, *bla*_CTX-M-15_, and *bla*_CTX-M-55_ were detected in 35, 5, and 5 isolates, respectively, with *bla*_SHV-2_ and *bla*_SHV-12_ both detected in one (differing) isolate (Table 1). The *bla*_OXA-10_ gene was concomitantly present with the *bla*_CTX-M-1_ gene in three isolates from chickens from House 2 (Table 1), while the *bla*_TEM_ gene remained undetected.

Among the 38 nalidixic-acid-resistant isolates, 21, 2, and 1 isolates harbored *aac(6′)*-Ib-*cr*, *qnr*S, and *qnr*B genes, respectively. The *aac(6′)*-Ib-*cr* gene was found in isolates from both houses (House 1: 18/25; House 2: 3/13); however, the *qnr*S and *qnr*B genes were only found in isolates from House 2, and they were associated with the *aac(6′)*-Ib-*cr* gene (Table 1). Among the 44 tetracycline-resistant isolates, the *tetB* gene (*n* = 26) was more prevalent than *tetA* (*n* = 14), with both *tet* genes found to co-occur in three isolates from House 2. Thirty-two isolates were sulfonamides-resistant and concurred with the presence of *sul*1 and *sul*2 genes in 29 and 18 isolates, respectively. Both genes were concomitantly detected in 15 isolates. The *mcr*-2 gene (the sequence is provided in the Supplementary Materials) was detected in two colistin-resistant *E. coli* isolates from ten resistant isolates (House 2 only), with *mcr*-1 not detected. Class 1 integron was detected in 29 isolates, while class 2 integron was detected in just five isolates (Table 1). Both integrons were concurrently present in one isolate (House 2). All *int*1-positive isolates amplified the conserved 3′ region (*qac*∆E-*sul*1).

### 3.3. Virulence Genes

The *fimH, fyuA, iutA,* and *papGIII* genes were detected in 47 (100%), 35 (74.4%), 23 (48.9%), and 17 (36.1%) ESBL-producing isolates, respectively. The *cnf1* gene was not detected. The *papGIII* gene was notably more prevalent among *E. coli* isolates from House 1 (*n* = 16; 55.1%) than House 2 (*n* = 1; 5.5%), while conversely, *iutA* was more prevalent among isolates from House 2 (*n* = 15; 83.3%) than House 1 (*n* = 8; 27.5%) (Table 1).

### 3.4. Phylogenetic Groups and Genetic Relatedness

**Figure 1:**
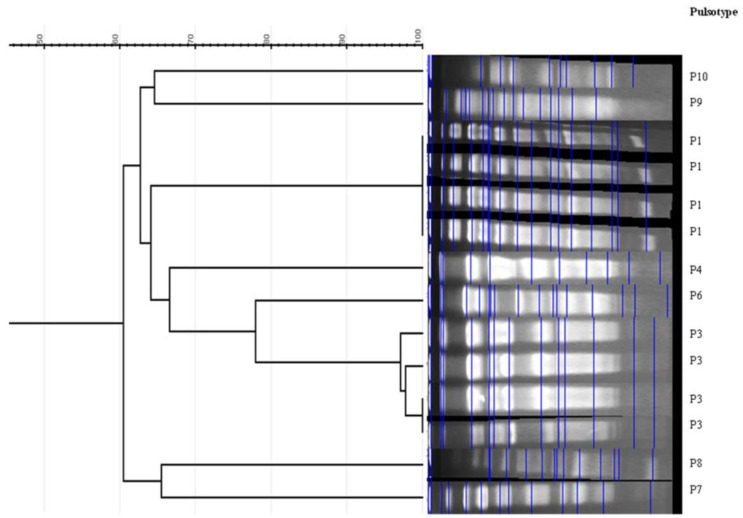
Representative PFGE cluster analysis of the main detected pulsotypes.

The majority of isolates belonged to phylogroup A (*n* = 41; 87.2%), with phylogroups F, C, and D represented by 3, 2, and 1 isolates, respectively. Four isolates were untypeable (DNA degraded) via PFGE, with the remaining 43 isolates classified into ten pulsotypes (P1 to P10) (Table 1, Figure 1). The predominant pulsotype P1 encompassed 23 isolates, all of which were collected from House 1. The remaining three typeable isolates in this house belonged to pulsotypes P4, P5, and P10. Conversely, isolates from House 2 exhibited a significantly higher level of diversity, with eight pulsotypes identified: pulsotypes P2, P3, P4, and P6 characterized 2, 5, 4, and 2 isolates, respectively. Pulsotypes P4 and P5 were shared by isolates collected from both Houses (Table 1). MLST was performed for 14 selected isolates, and the following STs were identified: ST224 (House 1/P1, 2 isolates), ST69 (House 1/P1, 1 isolate), ST410 (House 1 and 2/P4, 3 isolates; house 2/P2, 1 isolate), ST471 (House 1 and 2/P5, 2 isolates), ST617 (House 1/PFGE un-typeaple, 1 isolate), ST1642 (House 2/P3, 1 isolate), ST2460 (House 2/P6, 1 isolate), ST46 (House 2/P3, 1 isolate), and ST58 (House 2/PFGE un-typeable, 1 isolate) (Table 1).

## 4. Discussion

The current study collected 95 feces swab samples from two neighboring (≈300 M) chicken (*G. domesticus*)-rearing households in a rural area of northern Tunisia, 47 (49.4%) of which were colonized by ESBL-producing *E. coli* isolates. Chickens from House 1 exhibited a significantly higher percentage of colonization by ESBL-producing *E. coli* isolates than House 2 (29 (72.5%) versus 18 (32.7%) isolates). The high occurrence of ESBL-producing *E. coli* found in the current study mirrors the high rates reported from poultry farms employing intensive production methods, both in Tunisia and further afield [52,53,54]. For example, a recent study by Subramanya et al [55] reported that 19 out of 66 (28.8%) cloacal swab samples from healthy *G. domesticus* were ESBL positive, of which 16 isolates (16/66; 28.7%) were ESBL-producing *E. coli*. Conversely, Shoaib et al [56] employed nonselective media for *E. coli* isolation from 150 sick backyard chickens (free-range), identifying just 8 (5.3%) ESBL-producing *E. coli* isolates. The majority of isolates from the current study were MDR, a trait commonly reported among ESBL-producing *E. coli* irrespective of their origin [12,26,27,57]. The high rate of gut colonization by ESBL-producing *E. coli* in organically raised chickens was unexpected, considering the (owner-reported) absence of previous antibiotic usage. Additionally, residents from both sampled houses reported that antibiotics had not previously been used for other proximal animals (cows, owe, dogs, pet, and donkeys). Accordingly, the role of antibiotic use among sampled chickens as a driver of multidrug-resistant ESBL-producing *E. coli* was deemed unlikely, if not impossible; thus, other pathways for resistance must have been present. For example, in both houses, free-range chickens were frequently in contact with household members and (presumably) wild animals, such as birds and rats, which were potentially colonized with ESBL-producing *E. coli*. Indeed, notwithstanding consumption of kitchen waste and proximity to wastewater, in many rural communities, householders frequently defecate and/or urinate adjacent to areas where chickens move freely. As such, potential transmission of human- or animal-derived ESBL-producing *E. coli* to chickens is plausible. Similarly, free-range chickens are omnivorous and permitted to roam freely and consume wheat grains, a variety of insects, plant debris, and vegetable wastes. Previous studies have reported insects (e.g., flies and cockroaches) as important reservoirs for ESBL-producing *E. coli* in both urban and rural environments [58,59,60]. Similarly, several studies have reported contamination of vegetables and plant organic matter by ESBL-producing *E. coli* [61,62]. Given the complexity of antimicrobial resistance and its transmission within and between various ecological niches, it is extremely difficult to confidently trace and identify the origins of antimicrobial resistance acquisition. The limit of this study was the absence of analysis of samples from humans, soil, and animals in both houses; this would have provided important data about the reservoirs/vectors of the collected isolates.

An understanding of the epidemiology of genes encoding ESBL enzymes might be employed to indicate the possible origins of ESBL-producing *E. coli* isolates. In Tunisia, *bla*_CTX-M-1_ has been shown to represent the dominant gene encoding ESBL production in *E. coli* isolated from poultry samples [13,27,52,53], while *bla*_CTX-M-15_ typically predominates among human-derived ESBL-producing *E. coli* [63,64]. In the current study, *bla*_CTX-M-1_ was detected in 35 of 47 isolates, followed by *bla*_CTX-M-15_ (*n* = 5) and *bla*_CTX-M-55_ (*n* = 5). The *bla*_CTX-M-55_ gene has been frequently reported among livestock in Asian countries [65]; recent studies from Tunisia have reported increasing emergence among avian ESBL-producing *E. coli* [27], in addition to recently being found for the first time among *E. coli* isolates from urban wastewater samples [12,26]. Notably, and to the best of the authors knowledge, this is the first report of the presence of *bla*_OXA-10_, herein found in three isolates in concurrence with *bla*_CTX-M-1_, among ESBL-producing *E. coli* of animal origin. OXA-10 (PSE-2), belonging to class D β-lactamases, is not an ESBL enzyme but possesses the ability to hydrolyze cephalosporins, hydrolyzing cefotaxime, ceftriaxone, and aztreonam at low levels but sparing ceftazidime, cephamycins, and carbapenems [66]. This enzyme is primarily detected in ESBL-producing *Pseudomonas aeruginosa* and *Acinetobacter baumannii* isolates [67,68] but rarely from *E. coli* [69].

Colistin is one of the “last-resort” antimicrobial agents for treatment of infections caused by ESBL/plasmidic AmpC/Carbpenemase-producing *Enterobacteriaceae*. Over the past 5–7 years, acquired mobile colistin resistance (*mcr*) determinants have increasingly been reported worldwide, with at least 10 variants (*mcr*-1 to *mcr*-10) having been characterized [70]. In the current study, two of ten colistin-resistant isolates carried the *mcr*-2 gene, with *mcr*-1 remaining undetected. To date, while the *mcr*-1 and *mcr*-2 genes have never been reported in human-derived enterobacteria in Tunisia, *mcr*-1 has been increasingly reported among animal-derived ESBL-producing *E. coli*, including poultry [27,71], dairy cattle [72], camels [53], and wastewater treatment plants [26]. Accordingly, to the best of the authors’ knowledge, the current study is the first to report the occurrence of *mcr*-2 from animal origin in Tunisia.

In the current study, genetic determinants for tetracycline and sulfonamide resistance were predominantly encoded by the globally ubiquitous *tet*A/*tet*B and *sul*1/*sul2* genes, respectively [12,26,27,53,72]. With respect to plasmid-mediated quinolone resistance (PMQR) determinants, the *aac(6′)*-Ib-*cr* gene dominated, followed by *qnr*S and *qnr*B genes, all of which are frequently reported in ESBL-producing *E. coli* from various origins [5,12,26,27,72,73,74]. The aforementioned genes were primarily identified as being plasmid borne or a part of integron structures, which enhances their horizontal transfer within strains belonging to the same genera or between different enterobacteria genera. Class 1 integrons were detected in 27 isolates, whereas class 2 integrons were detected in just four isolates. The predominance of class 1 integrons has been well documented [12,25,26,27,72] and is almost certainly associated with the active integrase gene of class 1 integron, which has the ability to integrate several gene cassettes encoding antibiotic resistance in their variable regions [75].

Genetic relatedness determined by PFGE indicated a low level of isolate heterogeneity within each sampled household, albeit one analogous clone (pulsotype) being identified within each subsample. Within household 1, pulsotype P1 comprised 23 out of 29 isolates, with this clone not detected in household 2. Pulsotype P1 exhibited highly variable resistance profiles, antimicrobial resistance genes, virulence factors, and integron content, thus pointing to a high degree of genetic dynamicity. Three isolates of this pulsotype were types by MLST and revealed two isolates belonging to the ST224 (CTX-M-1/CTX-M-15) and one ST69 (CTX-M-55), which are known as pandemic or international high-risk clonal lineages. ST224 appears to be well adapted to the human–animal interface, being reported globally, including Tunisia (North Africa), mostly in association with plasmid-mediated *bla*_CTX-M_-type genes [13,76]. The clonal lineage ST69 (CTX-M-55-positive) has been reported in other studies from clinical, livestock, and environmental samples [12,13,77], and it is one of the most common lineages of extraintestinal pathogenic *E coli* [78]. In addition, the unique isolate of pulsotype P4 in household 1 was assigned to ST410, which is also known as a high-risk clonal lineage associated with both nosocomial and community-acquired infections and is being increasingly detected from multiple origins worldwide, including Tunisia [13,78]. However, the ST471 (pulosotype 5) and ST617 (untypable by PFGE) lineages have been rarely reported from human and animal origins and are mostly associated with ESBL or carabapenemase production [79,80]. Conversely, despite a lower number of studied isolates, isolates from household 2 were more heterogeneous, with four pulsotypes characterizing more than one isolate, all of them also concurrent with variable phenotypic and genotypic traits. In addition, six different STs were identified (ST46, ST58, ST410, ST471, ST1642, and ST2460). Significantly, ST410 and ST471 were shared by isolates collected from both houses, potentially indicating transmission of ESBL-producing *E. coli* between chickens reared in the two houses. This may be expected, since in this rural zone, contact between chickens and livestock from differing houses within the same neighborhood is relatively common. It is also plausible that chickens were contaminated by a common vector(s) or source(s) within the general vicinity. A sizeable majority of isolates belonged to phylogroup A (87.2%), followed by phylogroups F (*n* = 3), C (*n* = 2), and D (*n* = 1); however, no isolates from phylogroup B2 were identified. Several studies have shown that avian *E*. *coli* strains are primarily assigned to commensal phylogroups A and B1, with a majority of extraintestinal pathogenic *E*. *coli* isolates of human origin associated with phylogroup B2, and to a lesser degree, group D [81]. However, the detected virulence genes (*fyuA, fimH, papGIII,* and *iutA*) are commonly reported among both avian pathogenic *E. coli* (APEC) and extrapathogenic *E. coli* causing human infections [17,82], thus highlighting the zoonotic potential of these isolates to cause human infections, albeit additional virulence genes require future investigation to confirm this hypothesis.

## 5. Conclusions

The presented study highlights the significant prevalence of ESBL-*E. coli* isolates among free-range chickens, the predominance of *bla*_CTX-M-1_ gene, and the likely spread of two minor clones, ST410 and ST471, known as international high-risk clonal lineages, between two proximal subsamples within a rural area of northern Tunisia. However, since only limited chicken samples and rural houses were analyzed, it is impossible to deeply understand the real epidemiology of ESBL-producing *E. coli* isolates from free-range chickens in this area. Many factors can influence the occurrence of such isolates in these chickens, such as colonization of the residents from both sampled houses and animals (domestic and wild) in their vicinity. Therefore, the main limit of this study was the studying of chickens separately from their biotic and abiotic environment in order to accomplish the goal of the “One health” concept.

## Figures and Tables

**Table 1:** Phenotypic and genotypic characteristics of the 47 ESBL-producing *E. coli* isolates collected from free-range chickens.

Isolate	Phylogroup	PFGE	ST *	Resistance Profile to Non-β-Lactam Antibiotics	*bla* Genes	Other Genes Detected/Integrons	Virulence Genes
household 1 (29 isolates)							
EC20	A	P1	ST224	NAL, TET, SXT, SU	CTX-M-1	*aac(6′)-Ib-cr, tetB, sul1, sul2/int1*	*fyuA, fimH, papGIII,*
EC22	A	P1	-	NAL, TET, SXT, SU	CTX-M-1	*aac(6′)-Ib-cr, tetB, sul1, sul2/int1*	*fyuA, fimH*
EC23	A	P1	-	NAL, TET, SXT, SU	CTX-M-1	*aac(6′)-Ib-cr, tetB, sul1/int1*	*fyuA, fimH, papGIII,*
EC24	A	P1	-	NAL, TET, SXT, SU	CTX-M-1	*aac(6′)-Ib-cr, tetB, sul1, sul2/int1*	*fyuA, fimH*
EC26	A	P1	-	NAL, TET, SXT, SU	CTX-M-1	*aac(6′)-Ib-cr, tetB, sul1, sul2/int1*	*fyuA, fimH, papGIII*
EC27	A	P1	-	NAL, TET, SXT, SU	CTX-M-1	*aac(6′)-Ib-cr, tetB, sul1, sul2/int1*	*fyuA, fimH, papGIII*
EC28	A	P1	-	NAL, TET, SXT, SU, CS	CTX-M-1	*aac(6′)-Ib-cr, tetB, sul1, sul2/int1*	*fyuA, fimH*
EC29	A	P1	-	NAL, TET, SXT, SU	CTX-M-1	*aac(6′)-Ib-cr, tetB, sul1, sul2/int1*	*fyuA, fimH*
EC30	A	P1	-	NAL, TET, SXT, SU	CTX-M-1	*aac(6′)-Ib-cr, tetB, sul1/int1 + int2*	*fyuA, fimH, papGIII*
EC31	A	P1	-	NAL, CIP, TET	CTX-M-1	-/-	*fyuA, fimH, papGIII*
EC33	A	P1	-	NAL, TET, SXT, SU	CTX-M-1	*tetB, sul1/int1*	*fimH*
EC34	A	P1	-	NAL, TET, SXT, SU	CTX-M-1	*aac(6′)-Ib-cr, tetB, sul1/int1*	*fyuA, fimH, papGIII, iutA*
EC35	A	P1	-	NAL, TET, SXT, SU	CTX-M-1	*aac(6′)-Ib-cr, tetB, sul1/int1*	*fyuA, fimH, papGIII,*
EC36	A	P1	ST69	NAL, TET, SXT, SU	CTX-M-55	*tetB, sul1/int1*	*fyuA, fimH, papGIII*
EC37	A	P1	-	TET	CTX-M-1	*tetA*/-	*fimH, iutA*
EC38	A	P1	-	NAL, TET, SXT, SU	CTX-M-1	*aac(6′)-Ib-cr, tetB, sul1/int1*	*fyuA, fimH, papGIII*
EC39	A	P1	-	TET	CTX-M-1	*-*/-	*fimH, papGIII, iutA*
EC40	A	P1	-	NAL, CIP, TET, GEN, SXT, SU	CTX-M-1	*aac(6′)-Ib-cr, tetB, sul1/int1*	*fimH*
EC41	A	P1	ST224	NAL, CIP, TET	CTX-M-15	*tetA*/-	*fyuA, fimH, iutA*
EC43	A	P1	-	NAL, CIP	CTX-M-15	-/-	*fyuA, fimH iutA*
EC45	A	P1	-	NAL, TET, SXT, SU	CTX-M-1	*tetB, sul1/int1*	*fyuA, fimH, papGIII, iutA*
EC46	A	P1	-	NAL, CIP, SXT, SU	CTX-M-1	*aac(6′)-Ib-cr, sul1/int1*	*fyuA, fimH, papGIII*
EC47	A	P1	-	NAL, TET, SXT, SU	CTX-M-1	*aac(6′)-Ib-cr, tetB, sul1/int1*	*fyuA, fimH, papGIII*
EC42	A	P4	ST410	TET	CTX-M-15	*tetA/-*	*fimH, iutA*
EC25	F	P5	ST471	TET, SXT, SU	SHV-2	-/-	*fimH, iutA*
EC21	A	P10	-	NAL, TET, SXT, SU	CTX-M-1	*aac(6′)-Ib-cr, tetB, sul1, sul2/int1*	*fyuA, fimH*
EC19	A	NT	-	NAL, TET, SXT, CS	CTX-M-55	*tetB, sul1, sul2/int1*	*fyuA, fimH, papGIII*
EC32	C	NT	-	NAL, CIP	CTX-M-15	*aac(6′)-Ib-cr, sul1/int1*	*fyuA, fimH*
EC44	A	NT	ST617	NAL, TET, SXT, SU, CS	CTX-M-1	*aac(6′)-Ib-cr, tetB, sul1/int1*	*fyuA, fimH, papGIII*
household 2 (18 isolates)							
EC1	A	P2	ST410	NAL, TE, SXT, SU	CTX-M-1 + OXA10	*aac(6′)-Ib-cr, tetB, sul1, sul2/int1*	*fyuA, fimH, papGIII*
EC3	A	P2	-	NAL, TET, SXT, SU	CTX-M-1 + OXA10	*qnrS, tetA, sul2/-*	*fimH*
EC5	A	P3	ST1642	NAL, CIP, TET, SXT, SU	CTX-M-1	*tetA, tetB, sul1, sul2/int1*	*fyuA, fimH, iutA*
EC6	A	P3	-	NAL, CIP, TET, SXT, SU, CS	CTX-M-1	*tetA, sul1, sul2/int1*	*fyuA, fimH, iutA*
EC7	A	P3	ST46	NAL, CIP, TET, SXT, SU, CS	CTX-M-55	*tetA, sul1, sul2/int1*	*fimH, iutA*
EC8	A	P3	-	NAL, CIP, TET, SXT, SU, CS	CTX-M-1	*tetA, sul1, sul2, mcr-2/int1 + int2*	*fyuA, fimH, iutA*
EC10	A	P3	-	NAL, CIP, TET	CTX-M-1	*aac(6′)-Ib-cr qnrS, tetA/-*	*fyuA, fimH, iutA*
EC2	A	P4	ST410	NAL, CIP, TET, CS	CTX-M-1 + OXA10	*tetA, tetB, mcr-2/-*	*fyuA fimH, iutA*
EC12	A	P4	-	TET	CTX-M-55	*tetA/-*	*fimH, iutA*
EC16	A	P4	-	TET, CS	CTX-M-1	*tetA/-*	*fimH, iutA*
EC18	D	P4	ST410	NAL, CIP, TET, SXT, SU	CTX-M-1	*aac(6′)-Ib-cr, qnrB, tetA, tetB, sul2/int2*	*fyuA, fimH, iutA*
EC14	A	P5	ST471	TET, GEN, CS	CTX-M-1	*tetA/-*	*fyuA, fimH, iutA*
EC13	A	P6	ST2460	NAL, CIP, TET, CS	CTX-M-1	*tetA/-*	*fyuA, fimH, iutA*
EC17	A	P6	-	NAL, CIP, TET, CS	CTX-M-1	*tetA/-*	*fyuA, fimH, iutA*
EC4	C	P7	-	NAL, CIP, TET, GEN	CTX-M-1	*tetA, sul1/int1*	*fimH*
EC9	F	P8	-	TET, SXT, SU, CS	SHV-12	*tetB, sul2/int2*	*fyuA, fimH, iutA*
EC15	F	P9	-	NAL, CIP, TET, SXT, SU	CTX-M-15	*tetB/int2*	*fyuA, fimH, iutA*
EC11	A	NT	ST58	TET, SXT, SU, CS	CTX-M-55	*sul1, sul2/int1*	*fimH, iutA*

EC: *E*. *coli*, NAL: nalidixic acid, CIP: ciprofloxacin, SXT: trimethoprim/sulfamethoxazole; SU: sulfonamides, TET: tetracycline, GEN: gentamicin; CS: colistin, NT: not typeable, ST *: sequence type (MLST was performed for representative *E. coli* isolates).

## Data Availability

All data generated and analyzed during our study are included in this article.

## Supplementary Materials

The following supporting information can be downloaded at: https://www.mdpi.com/article/10.3390/genes14040875/s1, Sequence of mcr-2 gene identified in *Escherichi coli* EC2 and EC8 isolates. Identical to the sequence accession no: MW811416.
