# Supplementary material for: Occurrence of High-Risk Clonal Lineages ST58, ST69, ST224, and ST410 among Extended-Spectrum β-Lactamase-Producing Escherichia coli Isolated from Healthy Free-Range Chickens (Gallus gallus domesticus) in a Rural Region in Tunisia
Source: Genes (Basel). 2023 Apr 6;14(4):875. doi: 10.3390/genes14040875 (PMC10138121; doi:10.3390/genes14040875)
Supplement: Supplementary file 1 [file genes-14-00875-s001.zip › genes-2239965-supplementary.pdf]

Supplementary file

Sequence of mcr-2 gene identified in *Escherichi coli* EC2 and EC8 isolates. Identical to the sequence accession no: MW811416

1 tgttgcttgt gccgattggg ctatttagca gtcagtatgc gagtttcttt cgggtgcata
agccagtgcg tttttatatc aatccgatta cgccgattta ttcggtgggt aagcttgcca
gtatcgagta caaaaaagcc actgcgccaa cagacaccat ctatcatgcc aaagacgccg
tgcagaccac caagccgagc gagcgtaagc cacgcctagt ggtgttcgtc gtcggtgaga
cggcgcgtgc tgaccatgtg cagttcaatg gctatggccg tgagactttc ccgcagcttg
ccaaagttga tggcttggcg aattttagcc aagtgacatc gtgtggcaca tcgacggcgt
attctgtgcc gtgtatgttc agctatttgg gtcaagatga ctatgatgtc gataccgcca
aataccaaga aaatgtgcta gatacgcttg accgcttggg tgtgggtatc ttgtggcgtg
ataataattc agactcaaaa ggcgtgatgg ataagctacc tgccacgcag tattttgatt
ataaatcagc aaccaacaat acc 563
